# Deriving and refining atomic models in crystallography and cryo-EM: the latest *Phenix* tools to facilitate structure analysis

**DOI:** 10.1107/S2059798319013391

**Published:** 2019-10-01

**Authors:** Bruno P. Klaholz

**Affiliations:** a Centre for Integrative Biology (CBI), Department of Integrated Structural Biology, IGBMC, CNRS, Inserm, Université de Strasbourg, 1 rue Laurent Fries, Illkirch 67404, France; b Institute of Genetics and of Molecular and Cellular Biology (IGBMC), 1 rue Laurent Fries, Illkirch, France; c Centre National de la Recherche Scientifique (CNRS), UMR 7104, Illkirch, France; d Institut National de la Santé et de la Recherche Médicale (Inserm), U964, Illkirch, France; e Université de Strasbourg, Illkirch, France

**Keywords:** structural biology, atomic model refinement, crystallography, cryo electron microscopy, *Phenix*

## Abstract

In structural biology, deriving and refining atomic models into maps obtained from X-ray crystallography or cryo electron microscopy (cryo-EM) is essential for the detailed interpretation of a structure and its functional implications through interactions so that for example hydrogen bonds, drug specificity and associated molecular mechanisms can be analysed. This commentary summarizes the latest features of the Phenix software and also highlights the fact that cryo-EM increasingly contributes to data depositions in the PDB and EMDB.

Over the last few years, the technical breakthrough of single-particle cryo electron microscopy (cryo-EM) thanks to the recent developments of direct electron detectors and advanced image processing software (for reviews see, for example, Orlova & Saibil, 2011 ▸; Stark & Chari, 2016 ▸; Orlov *et al.*, 2017 ▸; Kim *et al.*, 2018 ▸; Ognjenović *et al.*, 2019 ▸), has in many cases allowed the 3 Å resolution range to be reached (Fig. 1 ▸). In X-ray crystallography, this resolution range is generally considered to be required for deriving atomic models with properly defined geometrical properties, such as the peptide backbone and side-chain geometry of amino acids and nucleotides in macromolecular complexes. The reason why deriving reliable atomic models is so important is that it provides the basis for the detailed analysis of the three-dimensional structures of interest, such as nucleoprotein complexes in the cell nucleus, membrane proteins or viruses and various drug targets. An atomic model containing flaws because of incorrect or insufficient refinement may lead to incorrect conclusions on the detailed interpretation of the structure, with direct implications on the analysis of interactions between residues. For example, the accurate description of hydrogen bonds (which relies both on proper distances and angular orientations of the acceptor and donor, *e.g.* Klaholz & Moras, 2002 ▸; Coulocheri *et al.*, 2007 ▸) are directly relevant for the analysis of molecular recognition or catalysis events, specificity of drug interactions, effects of point mutants *etc.*, and the precise depiction of base-pairing and stacking interactions between nucleotide bases is essential for the analysis of RNA and DNA complexes (Leontis & Westhof, 2001 ▸).

Three-dimensional maps obtained from X-ray diffraction describe the spatial electron-density distribution, while cryo-EM maps are electrostatic potential (ESP) maps as a result of the charged nature of the electrons used to image the biological sample. This difference can have important implications for the analysis of charged residues and ions (Wang & Moore, 2017 ▸; Hryc *et al.*, 2017 ▸; Wang *et al.*, 2019 ▸), but the general properties of these maps are similar such that analogous tools can be used to build and refine atomic models into them (Fig. 1 ▸). In the X-ray crystallography field, a series of software packages are available (see, for example, https://www.rcsb.org/pdb/static.do?p=software/software_links/crystallography.html), some of which have been more specifically adapted to allow them to be used on cryo-EM maps [*e.g.* *REFMAC* (Brown *et al.*, 2015 ▸), *Buster* (Smart *et al.*, 2012 ▸) or *Phenix* (Afonine, Poon *et al.*, 2018 ▸), see also overall procedures described in Natchiar *et al.* (2017*b* ▸)], while others originated more from the cryo-EM field [see review by Malhotra *et al.* (2019 ▸), and references therein; Pintilie & Chiu (2018 ▸)]. This commonly involves real-space refinement because the cryo-EM maps can be used to refine the atomic model directly, without modifying the map (*i.e.* they intrinsically comprise experimental phases). ‘Structure’ refinement in cryo-EM means a map refinement that primarily comprises particle centring (translation and rotation) and Euler angle assignment to iteratively improve the 3D reconstruction (technically, this is a back-projection obtained from individual 2D particle views), but once the map is fully refined, it is not modified further apart from applying a high-pass filter to better visualize high-resolution features (if present, otherwise this only increases the noise level). By contrast, for diffraction data the map is iteratively modified and refined using phase information derived from the atomic model that is under refinement (phase information can also come from experimental phasing, such as native sulfur phasing, SAD, MIR *etc.*, but it rarely extends to high resolution and it often needs to be combined with model-derived phases). In the cryo-EM field, various software has been developed in the past for the analysis of low to medium resolution maps (including flexible fitting *etc.*), which is not the focus of this commentary because specifically for high-resolution maps (regardless of whether they come from crystallography or cryo-EM), it is essential to refine the detailed geometry of the model and validate it for data deposition into the appropriate databases (PDB, EMDB and associated databases) to which cryo-EM is increasingly contributing (Fig. 1 ▸).

Here, we comment on the article by Liebschner *et al.* in this issue of *Acta Cryst. D* (Liebschner *et al.*, 2019 ▸), which describes the various tools available in the software suite *Phenix*, including the most recent developments, thus providing a comprehensive and extensive description of the latest version. The major aim is to provide any user with informatics tools, including robust default settings, which allow a high level of automation. This is not only to simplify the work, but also contributes to reducing errors in refinements because iteration between automatic refinement and manual model building/validation are often required. The article addresses the challenge of listing and briefly describing all the main parts of the program suite, which can handle X-ray and neutron diffraction data, and cryo-EM data. The article will be useful for any reader, specialist or newcomer: it gives an overview of the structure determination steps, the specifics of structure determination from X-ray and neutron diffraction data such as crystal twinning analysis, native Patterson functions, SAD phasing and related methods based on the presence of an anomalous signal, molecular replacement *etc.*, refinement of atomic models into maps obtained using diffraction or cryo-EM methods, and new specific tools for cryo-EM map interpretation. As a software package, *Phenix* integrates all of these aspects, which is a major achievement and is very helpful for the community. As a suggestion to both the crystallography and cryo-EM fields, and considering that the resolution levels reached in cryo-EM nowadays allow the derivation of detailed atomic models, one should probably be more cautious with regards to the confusing usage of the term ‘model’, which implies the *atomic* model (the model being built into the map), while in the cryo-EM field the term is often used to mean the cryo-EM map itself. A suggestion would be to specify ‘atomic’ when we speak about atomic models in general, and in cryo-EM avoid the term ‘model’, instead using the terms cryo-EM map or 3D reconstruction, for example in the name of software subroutines (this can be an initial map or a refined map depending on the refinement stage during the structure determination process; this involves no atomic models unless they are used as the initial reference in the form of a calculated map that is low-pass filtered).

Several new tools, some coming from external developments, are integrated or interfaced with *Phenix*, for example *phenix.dock_in_map*, *CryoFit* and *ISOLDE* for flexible fitting, and *Pathwalker* to trace the backbone, which all help in building, refining and validating atomic models, *e.g.* with *phenix.mtriage* there are tools to estimate resolution (*d*_99_) or to calculate map-model Fourier correlation curves (Afonine, Klaholz *et al.*, 2018 ▸). However, for disordered regions it can be difficult to build a reliable atomic model, in which case the presence of flexible structures needs to be addressed, *e.g.* by ensemble refinement (Burnley *et al.*, 2012 ▸). In cryo-EM, various particle-sorting methods [based on 2D or 3D classification methods using multi-variate statistical analysis or maximum-likelihood approaches (Klaholz *et al.*, 2004 ▸; White *et al.*, 2004 ▸; Penczek *et al.*, 2006 ▸; Orlova & Saibil, 2010 ▸; Scheres, 2010 ▸; Lyumkis *et al.*, 2013 ▸; Klaholz, 2015 ▸; Serna, 2019 ▸)] have been developed to separate different structures, describe several conformational states and address the dynamics of macromolecular complexes. The maps of the particle sub-populations that describe a similar conformation can then be further refined using focused classifications and specific refinements to reach a high-resolution for the entire complex (Ilca *et al.*, 2015 ▸; von Loeffelholz *et al.*, 2017 ▸; Nakane *et al.*, 2018 ▸) for which *Phenix* provides a tool for assembling a weighted composite map from the refined sub-regions. As the resolution is often not constant throughout a cryo-EM map (the concept of local resolution; Cardone *et al.*, 2013 ▸; Kucukelbir *et al.*, 2014 ▸) there is a tool for local filtering (*phenix.auto_sharpen*), which uses the current atomic model taking into account the atomic displacement (*B*) factors, similarly to *LocScale* (Jakobi *et al.*, 2017 ▸); however, the recent software *LocalDeblur* does not use an atomic model (Ramírez-Aportela *et al.*, 2019 ▸). To reflect a certain degree of flexibility, it is important to also refine temperature factors for cryo-EM derived atomic models (Wlodawer *et al.*, 2017 ▸; usually a restrained *B*-factor refinement of all the atoms in an amino acid, to reduce the number of parameters to be refined). Moreover, including hydrogen atoms in the final atomic model refinements can also improve the clash score for cryo-EM data (Orlov *et al.*, 2019 ▸). The *Phenix* graphical user interface (GUI) is interfaced with the graphics programs *Coot* (Emsley *et al.*, 2010 ▸) and *Pymol* (DeLano, 2002 ▸) to facilitate switching between automatic and manual refinement modes (*e.g.* for checking backbone Cα atom positions, flipping backbone peptides to cure Ramachandran plot outliers, correcting side-chain conformations, validating the entire structure *etc.*) and for performing detailed structure analysis, which is the original aim of a structural biology project. Finally, a convenient feature is also the possibility to prepare a table summarizing statistics for the structure determination and the geometrical parameters of the atomic model in crystallography or cryo-EM, together with the validation report linked with the wwPDB (https://www.wwpdb.org/validation/validation-reports). As for other tools, there is also a specific ‘bulletin board’ mailing list and an online tutorial (see http://www.phenix-online.org/mailman/listinfo/phenixbb and https://www.youtube.com/c/phenixtutorials). Taken together, the latest features of *Phenix* are not only convenient for full workflows but also respond to specific needs, depending on the applications and user expertise.

Clearly, the next challenge will be to integrate atomic model building into large-scale approaches, particularly in cryo electron tomography, which when combined with sub-tomogram averaging can provide maps in the 30–10 Å resolution range and in exceptional cases that comprise internal symmetry even up to the 3–4 Å resolution range (Schur *et al.*, 2016 ▸). For this, various medium-resolution tools exist (including those in *Phenix*) and will need to be developed further, illustrating the ongoing move of the field towards multi-scale and multi-resolution, and correlative approaches to *in situ* macromolecular complexes (Orlov *et al.*, 2017 ▸; Jun *et al.*, 2019 ▸; Schaffer *et al.*, 2019 ▸). This includes super-resolution fluorescence imaging (nowadays single-molecule localization microscopy, SMLM, is also feasible in 3D, see for example Andronov *et al.*, 2018 ▸, 2019 ▸) to integrate all scales and achieve cellular structural biology in the future.

## Figures and Tables

**Figure 1:**
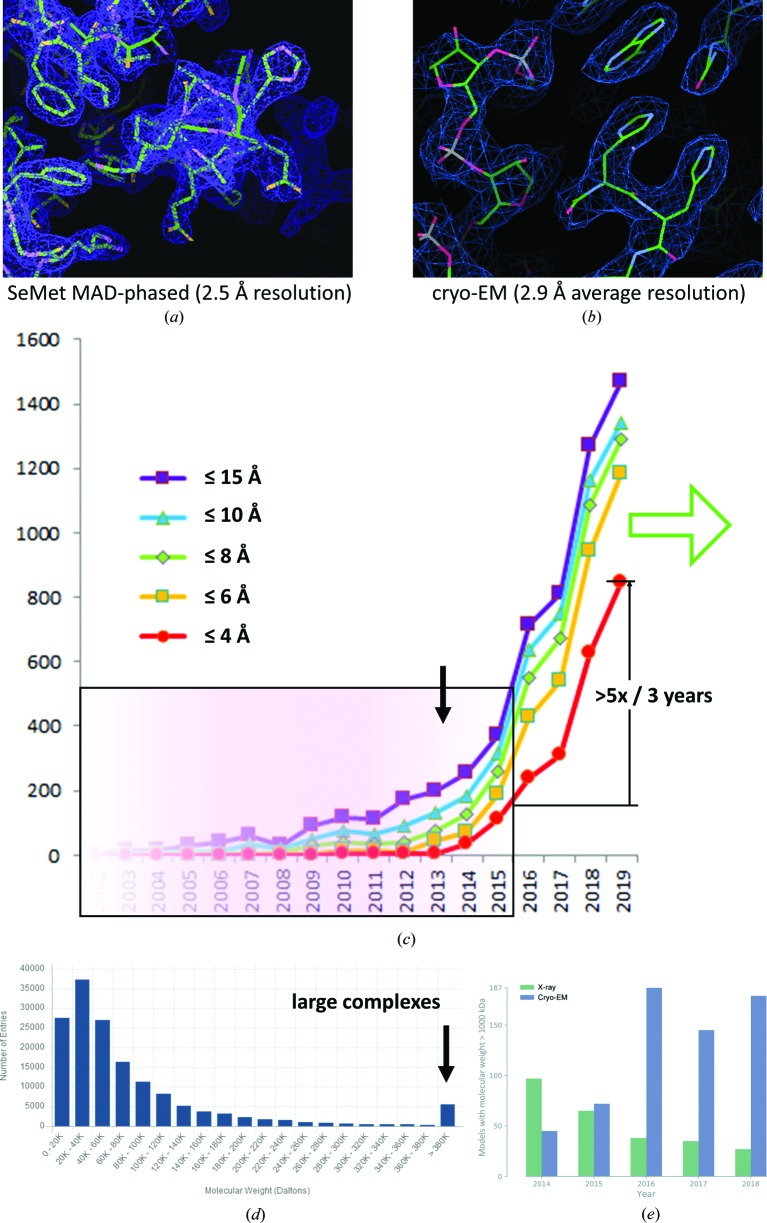
(*a*) X-ray crystallography electron-density map: SeMet (seleno­methio­nine) MAD (multiple-wavelength anomalous dispersion), phased at 2.5 Å resolution (translation initiation factor 2, IF2; PDB ID 4b3x; Simonetti *et al.*, 2013 ▸). Note the histidine residue defined by the map. (*b*) Cryo-EM map: obtained by 3D reconstruction from individual 2D particle images (60S ribosomal subunit of the 80S human ribosome, 2.9 Å average resolution with local resolution extending this; Natchiar *et al.*, 2017*a* ▸). Note the histidine residues defined by the map (to be compared with panel *a*) and the nucleotides in the vicinity. (*c*) Increasing number of cryo-EM maps deposited in the EMDB and achieving the specified resolution levels. The data are taken from the http://www.ebi.ac.uk/pdbe/emdb/ and http://www.rcsb.org/pdb/ websites [updated graph compared with the one shown in Orlov *et al.* (2017 ▸) as of 26 September 2016, within the shaded light pink box; and as of 20 September 2019], illustrating an over fivefold increase in cryo-EM structures at high resolution (4 Å or better, red curve) within the last three years. The black arrow marks the year 2013 where high-sensitivity detectors entered the cryo-EM field. (*d*) Graph showing the PDB data distribution by molecular weight. While most structures lie below 60 kDa and are determined by X-ray crystallography, those at high molecular weight (right-hand end) are more amenable to cryo-EM, although complexes in the 50–150 kDa range can now be targetted by cryo-EM as well. (*e*) Graph from Liebschner *et al.* (2019 ▸) in this issue, illustrating that since 2015, cryo-EM depositions have accounted for the majority of large macromolecular structures currently in the PDB.
